# Morphology Controlled Synthesis of γ-Al_2_O_3_ Nano-Crystallites in Al@Al_2_O_3_ Core–Shell Micro-Architectures by Interfacial Hydrothermal Reactions of Al Metal Substrates

**DOI:** 10.3390/nano11020310

**Published:** 2021-01-26

**Authors:** Dohyeon Han, Doohwan Lee

**Affiliations:** Department of Chemical Engineering, The University of Seoul, Dongdaemun-gu, Seoulsiripdaero 163, Seoul 02504, Korea; handh29@uos.ac.kr

**Keywords:** boehmite, alumina, morphology control, hydrothermal synthesis, core–shell structure, heterogeneous catalysts

## Abstract

Fine control of morphology and exposed crystal facets of porous γ-Al_2_O_3_ is of significant importance in many application areas such as functional nanomaterials and heterogeneous catalysts. Herein, a morphology controlled in situ synthesis of Al@Al_2_O_3_ core–shell architecture consisting of an Al metal core and a porous γ-Al_2_O_3_ shell is explored based on interfacial hydrothermal reactions of an Al metal substrate in aqueous solutions of inorganic anions. It was found that the morphology and structure of boehmite (γ-AlOOH) nano-crystallites grown at the Al-metal/solution interface exhibit significant dependence on temperature, type of inorganic anions (Cl^−^, NO_3_^−^, and SO_4_^2−^), and acid–base environment of the synthesis solution. Different extents of the electrostatic interactions between the protonated hydroxyl groups on (010) and (001) facets of γ-AlOOH and the inorganic anions (Cl^−^, NO_3_^−^, SO_4_^2−^) appear to result in the preferential growth of γ-AlOOH toward specific crystallographic directions due to the selective capping of the facets by adsorption of the anions. It is hypothesized that the unique Al@Al_2_O_3_ core–shell architecture with controlled morphology and exposed crystal-facets of the γ-Al_2_O_3_ shell can provide significant intrinsic catalytic properties with enhanced heat and mass transport to heterogeneous catalysts for applications in many thermochemical reaction processes. The direct fabrication of γ-Al_2_O_3_ nano-crystallites from Al metal substrate with in-situ modulation of their morphologies and structures into 1D, 2D, and 3D nano-architectures explored in this work is unique and can offer significant opportunities over the conventional methods.

## 1. Introduction

Chemical reactions on heterogeneous catalysts are complex and often coupled with the intrinsic surface reaction kinetics along with the mass and heat transport phenomena [1]. For the supported metal catalysts that are extensively used in the chemical industries, the intrinsic catalytic activities are mainly governed by atomic composition, crystal sizes, and exposed crystal-facets of the active metals and their interactions with supports [2,3]. The supports that immobilize active metal nanoparticles (NPs) on the surface necessitate high surface area and physicochemical stability. Meanwhile, the mass and heat transport through the heterogeneous catalysts are largely affected by the support properties such as thermal conductivity, surface area and porosity, and fluid-dynamics of reactants and products through the catalysts. Therefore, the effective design of the structures and selection of materials are of marked importance to achieve the high overall performance of heterogeneous catalysts in many industrial chemical reaction processes. Among various metal oxides, alumina is the most extensively utilized catalyst support because of its high physicochemical and mechanical properties and cost-effectiveness [4]. However, the majority of metal oxides including alumina have very low intrinsic thermal conductivities and high thermal capacities (thermal mass) that are adversary for delivering uniform temperature distribution and facile thermal conduction through the catalysts. Heat and mass transport throughout solid catalysts play significant roles in determining the efficiency and product selectivity of many industrial catalytic reactions and the life-time of the catalysts [5,6,7]. As shown in Table 1, the intrinsic thermal conductivity and specific heat capacity of Al_2_O_3_, SiO_2_, and TiO_2_ (extensively used materials for heterogeneous catalysts) are about one order magnitude inferior to the metals such as Al and Cu in terms of heat transport properties. The common approach to overcome this drawback has been the use of metallic foams on which non-active high surface area metal oxides are initially deposited followed by the introduction of the desired active metals or metal oxides [8]. However, the deep-coating approach is hard to apply for small catalyst particles and pellets of the common industrial catalysts forms, and the resulting structures are prone to degradation at the coated interface.

Previously, we reported a highly effective new design approach of heterogeneous catalysts and supports, constructing a core–shell composite architecture employing porous alumina (Al_2_O_3_) and MeAl_2_O_4_ spinel (Me = Ni, Cu, Co, Mg, Zn, etc.) from Al metal substrates [11,12]. This metal–ceramic core–shell architecture can provide high heat and mass transport properties along with significant surface properties, which are hardly achievable by the conventional heterogeneous catalysts. The Al@Al_2_O_3_ core–shell microarchitectures with Al-core encapsulated by the high surface area Al_2_O_3_ shell can be constructed by hydrothermal reactions (HTR) of Al particles. This synthesis approach is simple, unique, and scalable as the Al particles are used as the sole Al source for the porous alumina and the template of the core–shell architecture at the same time. The marked effectiveness of the Al@Al_2_O_3_ core–shell supports was demonstrated extensively with the applications in the highly endothermic methane reforming reaction [13] and the exothermic preferential CO oxidation in hydrogen-rich stream [14] under various practical operation conditions. These experimental studies revealed a significant increase in the performance of the core–shell catalysts largely owing to the facile heat and mass transport through the Al@Al_2_O_3_ core–shell supported catalysts.

In this work, we report in-situ modulation of the morphology and intrinsic properties of γ-Al_2_O_3_ nano-crystallites fabricated on Al metal particles in the Al@Al_2_O_3_ core–shell microstructures. The morphology, size, and structure of porous Al_2_O_3_ crystallites are of marked importance in heterogeneous catalysts as the dispersion and stability of active metal nanoparticles, surface acid–base properties, and mass transport properties of the catalysts are largely attributed to these intrinsic support properties. Several studies have reported the modulation of morphology and size of γ-Al_2_O_3_ by the controlled synthesis of boehmite (γ-AlOOH) and their transformation to γ-Al_2_O_3_ by calcination. The previous studies, however, mostly rely on sol–gel or hydrothermal synthesis using aluminum salts or alkoxides as the precursors in homogeneous solution [15,16]. Differently, the in-situ construction and modulation of the morphology and structure of Al@Al_2_O_3_ core–shell architectures by HTR of Al metal particles is based on the interfacial dissolution–reconstruction chemistry of Al metal in aqueous solution, which can be controlled by the presence of heteroatom ions, solution pH, and temperature. As will be discussed, γ-AlOOH nano-crystallites can be grown directly on an Al metal surface with various morphological features constructing the overall Al@Al_2_O_3_ core–shell microarchitecture, thereby providing unique and significant properties applicable for heterogeneous catalysts. The unique synthesis approach of Al_2_O_3_ nanostructures via heterogeneous dissolution and reconstruction chemistry of Al metal at the metal/solution interface and the resulting metal-ceramic core–shell microarchitecture can provide significant properties and opportunities over the conventional synthesis approaches and materials.

## 2. Materials and Methods

### 2.1. Materials

The pristine aluminum powder (Φ ≤ 25 µm, 99.9%) used for the synthesis was obtained from Goodfellow Co. (Huntingdon, UK) and used without any further treatment. Ammonium chloride (NH_4_Cl, 98.5%, Duksan chemical, Ansan, Korea), ammonium nitrate (NH_4_NO_3_, 99%, Junsei Chemical Co., Tokyo, Japan), and ammonium sulfate ((NH_4_)_2_SO_4_, 99%, Kanto chemical, Tokyo, Japan) were used for the preparation of aqueous solutions of heteroatom anions. Hydrochloric acid (HCl, 35–37%, Samchun chemical, Pyeongtaek, Korea), sulfuric acid (H_2_SO_4_, 95%, Samchun chemical, Pyeongtaek, Korea), acetic acid (CH_3_COOH, 99.7%, Alfa Aesar, Ward Hill, MA, USA), and ammonium hydroxide (NH_4_OH, 28%, Alfa Aesar, Ward Hill, MA, USA) were used to adjust the pH of the synthesis solution. All the chemicals in this work were used as received without any further purification or treatments.

### 2.2. Morphology Controlled Synthesis of Al@Al_2_O_3_ Core–Shell Microstructures

**Scheme 1:**
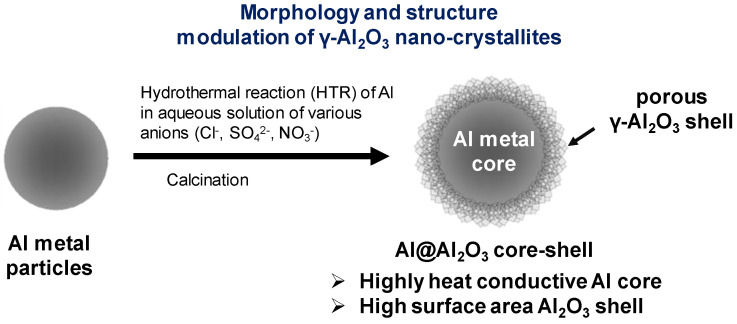
The Al metal-templated synthesis of Al@Al_2_O_3_ core–shell micro-architecture with modulation of the surface morphology of Al_2_O_3_ nanocrystallites by hydrothermal reactions (HTR) of Al metal particles in aqueous solution of heteroatom anions.

Scheme 1 describes the synthesis procedure for Al@Al_2_O_3_ core–shell microarchitecture with controlled morphology and structure of γ-Al_2_O_3_ nano-crystallites formed on the Al surface. Briefly, hydrothermal reactions of Al metal particles in H_2_O or an aqueous solution of heteroatom ions leads to the dissolution of Al atoms from the Al substrate and reconstruction into boehmite nano-crystallites at the Al-metal/solution interface, thereby constructing the Al@AlOOH core–shell microstructure. Subsequent calcination of the Al@AlOOH at elevated temperatures results in a topotactic transformation of the γ-AlOOH crystallites to porous γ-Al_2_O_3_, resulting in the final Al@Al_2_O_3_ core–shell microstructures. The typical synthesis procedure is as follows. First, the pristine aluminum powder (Goodfellow, 2.5 g) and deionized (D.I.) water (60 mL) were filled in a Teflon-lined autoclave. The hydrothermal reaction (HTR) was performed at 423 K and 473 K under autogenous pressure for 5 h. Next, the autoclave was cooled down to room temperature. The resultant samples were washed with a copious amount of water, dried at 393 K overnight, and calcined in a muffle furnace at 873 K for 4 h (ramp = 10 K min^−1^).

In order to investigate the effects of solution pH on the morphology and structure of Al@AlOOH core–shell, HTR of Al was conducted in aqueous solutions of NH_4_OH, HCl, and CH_3_COOH, respectively. The basic NH_4_OH solution (60 mL) was prepared at the pH of 10.5. The acidic HCl and CH_3_COOH solutions were prepared by adding HCl and CH_3_COOH in each 60-mL volume of D.I. water at the solution pH of 3.0 and 3.3, respectively. After adding Al powder (2.5 g) into the solutions, HTR was conducted at 423 K for 5 h.

HTR of Al powder was also conducted in aqueous solutions of various hetero-atom salts (NH_4_Cl, NH_4_NO_3,_ and (NH_4_)_2_SO_4_) to investigate the effects of the types of heteroatom anions on the morphology and structure of boehmite nanocrystallites. The heteroatom solutions were prepared dissolving NH_4_Cl (3.21 g), NH_4_NO_3_ (4.80 g), and (NH_4_)_2_SO_4_ (7.93 g) in each 60-mL volume of D.I water, respectively. The initial pH of the solutions was in the range of 6.2–6.4, and no additional pH adjustment was made. After adding the pristine Al powder (2.5 g), the hydrothermal reaction was conducted at 423 K for 5 h.

In addition, the HTR of Al was conducted in aqueous solution of (NH_4_)_2_SO_4_ with fine adjustments of the initial pH of the solution at 4 and 10, respectively. The (NH_4_)_2_SO_4_ solution was prepared as described above, and the solution pH was adjusted by dropwise addition of 0.1 M H_2_SO_4_ or 0.1 M NH_4_OH solution, respectively, under vigorous stirring of the solution with in-situ pH measurement using a pH meter (HI 2210, HANNA Instrument). The subsequent drying and calcination processes were kept the same as afore described. The synthesis of all the samples was repeated at least three times in order to verify reliability of the synthesis method and reproducibility of the resulting Al@AlOOH core–shell microstructures.

### 2.3. Characterizations

The surface morphologies of samples were characterized using a field emission scanning electron microscope (FE-SEM, SU8010, HITACHI) at an acceleration voltage of 2 kV. The X-ray diffraction (XRD) patterns of the samples were obtained (scan rate = 0.05° min^−1^) using a spectrometer (SmartLab, Rigaku) with monochromic Cu-Kα radiation operated at 3 kW. The N_2_ adsorption–desorption isotherm of the samples was obtained in a volumetric unit (Tristar II, Micromeritics) after degassing under a vacuum at 523 K for 4 h. The specific surface area and pore size distribution of the samples were characterized from the isotherms by the Brunauer–Emmett–Teller (BET) and Barrett–Joyner–Halenda (BJH) methods, respectively. The total pore volume of the samples was obtained by single point adsorption at the saturation pressure. Zeta potential measurement of the samples was carried out using an analyzer (ELS-1000ZS, Otsuka Electronics) at various solution pHs in the range from 3 to 11.

## 3. Results

### 3.1. Morphology and Structural Properties of Al@Al_2_O_3_ Core–Shell Prepared by HTR in D.I.-H_2_O

**Figure 1:**
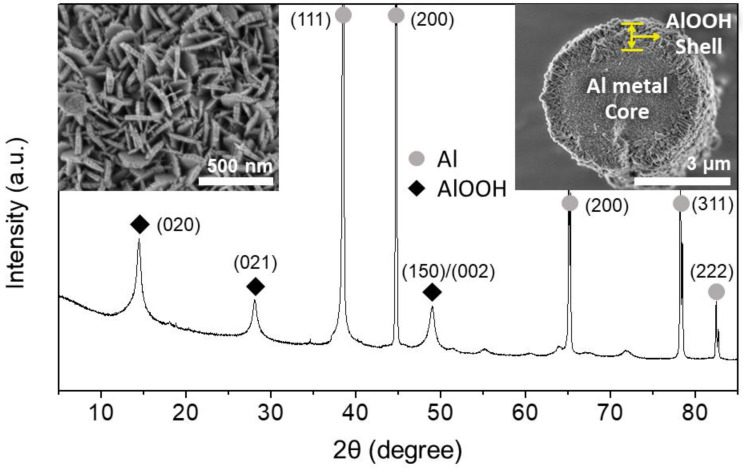
XRD pattern and SEM micrographs (insets: surface morphology (**left**) and cross-section (**right**)) of the Al@AlOOH core–shell prepared by HTR of Al metal particles at 373 K for 5 h.

Figure 1 shows the typical XRD pattern, SEM micrographs, and the cross-section of the Al@AlOOH core–shell architecture obtained by HTR (373 K, 5 h) of Al particles in D.I. water. The superposition of the characteristic XRD peaks of Al metal and boehmite crystallites indicates the construction of the Al–AlOOH composite structure by HTR of Al metal particles. The peaks appearing at 2θ of 38.5, 44.7, 65.1, 78.2 and 82.5° are assigned to the diffraction on the (111), (200), (220), (311) and (222) crystal planes of Al metal (Inorganic Crystal Structure Database (ICSD) #240129). The peaks at 14.5, 28.1, and 48.9° are assigned to the diffraction on the (020), (021) and (150)/(002) planes of boehmite crystallite (ICSD #100390). The average crystal size of the boehmite estimated by Debye–Sherrer equation from the (020) peak of the sample was 12 nm. The SEM micrograph of the cross-section of the sample reveals the construction of core–shell micro-architecture with an approximate thickness of the γ-AlOOH shell of 410 nm on average. The SEM micrographs of the surface of Al@AlOOH core–shell shows densely grown boehmite nano-crystallites on the Al surface, constructing the overall shell structure. A close examination of the surface indicates that these individual boehmite nano-crystallites have a thin platelet-like morphology with an average thickness of 26 nm and a width of 55–330 nm. The shapes and sizes of these crystallites were highly uniform, and the overall morphology of the γ-AlOOH shell was flower-like with the boehmite nano-platelets resembling petals with their edges perpendicular to the surface of the Al metal surface. The packing of these AlOOH nano-platelets was random without any preferential orientation parallel to the Al surface. The results collectively show that the Al@AlOOH core–shell microarchitecture can be formed in-situ by a simple interfacial HTR of Al metal particles, utilizing Al substrate as the sole metal precursor and a template for the final metal–ceramic composite structures.

**Figure 2:**
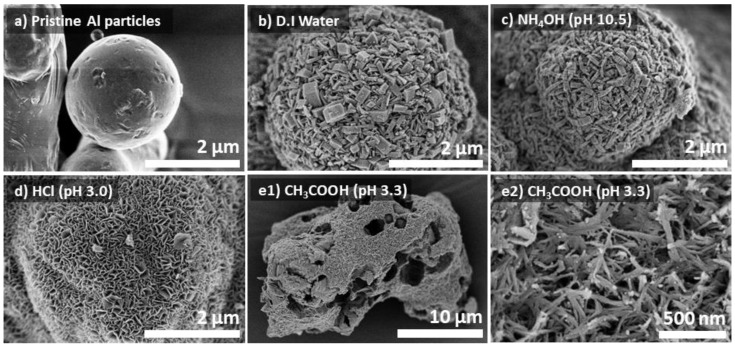
SEM micrographs of the pristine Al particles (**a**) and the Al@AlOOH core–shell structures prepared by HTR in D.I.-H_2_O (**b**), and aqueous solution of NH_4_OH (**c**) and HCl (**d**). The sample prepared in aqueous solution of CH_3_COOH (**e1**,**e2**) did not show the construction of a Al@AlOOH core–shell structure.

Aluminum hydroxide is amphoteric with increasing solubility in an aqueous solution both in acidic and basic conditions [17]. Therefore, the synthesis of Al@AlOOH was conducted at various initial pHs of the solution. Figure 2 displays SEM micrographs of the pristine Al and Al@AlOOH core–shell samples prepared in D.I.-H_2_O (pH = 7.2) and aqueous solutions of NH_3_OH (pH = 10.5), HCl (pH = 3.0), and CH_3_COOH (pH = 3.3), respectively. The samples were obtained by HTR at 423 K for 5 h. The SEM micrograph shows the sphere geometry with smooth surface of the pristine Al metal particles (Figure 2a). On the contrary, the sample obtained by HTR of the Al metal particles in H_2_O (Figure 2b) exhibited the presence of a number of boehmite nano-crystallites grown densely on the Al surface. The morphology, size, and spatial orientation of the crystallites were much less uniform compared to those grown at the low temperature of 373 K (Figure 1). The shapes of these boehmite crystallites resembled platelets or cuboids, and their sizes and thickness varied from several tens to hundreds of nanometers. Overall, the platelets were very thick with an approximate width/thickness (W/T) aspect ratio of 1.9. Differently, the Al@AlOOH prepared under the basic condition (pH = 10.5, Figure 2c) demonstrated uniform surface morphology and size of the boehmite nano-crystallites. The thickness of the γ-AlOOH crystallites fell between 30 and 120 nm and their W/T aspect ratio was about 4.1, which was about twice as large as that prepared under the neutral pH condition (Figure 2b). Markedly different surface morphologies of the samples were obtained when they were prepared in acidic conditions (pH = 3.3) in aqueous solutions of HCl and CH_3_COOH, respectively. The sample prepared in the HCl solution displayed extremely thin γ-AlOOH platelets with an average thickness of 11 nm grown on the Al particles, constructing the Al@AlOOH core–shell microarchitecture (Figure 2d). The W/T aspect ratio of these platelets was large at ~24, and the crystallites showed a unidirectional spatial orientation with their edges perpendicular to the Al surface, constructing the flower-like surface morphology. However, such a core–shell architecture was not developed when the CH_3_COOH solution was used even at the same initial pH condition. As can be seen clearly, the sample prepared in the aqueous solution of acetic acid exhibits random and destructed forms (Figure 2e1), differing largely from the typical core–shell geometry of Al@AlOOH samples. The morphology of these crystallites was random with coagulated fiber-like structures. These results collectively suggest the significant effects of the heteroatom anions in the synthesis solution on the growth characteristics of boehmite crystallites at the Al-metal/solution interface during HTR.

### 3.2. The Effects of Heteroatom Anions on the Interfacial Growth of Boehmites Crystallites

**Figure 3:**
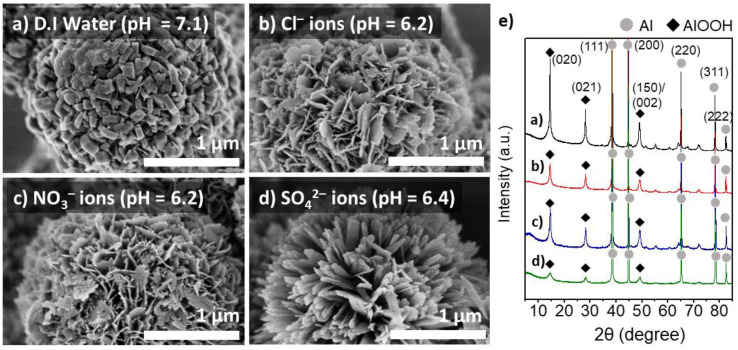
SEM micrographs of the Al@AlOOH core–shell samples prepared by HTR at 423 K for 5 h in D.I.-H_2_O (**a**) and the aqueous solution of NH_4_Cl (**b**), NH_4_NO_3_ (**c**), and (NH_4_)_2_SO_4_ (**d**), respectively. The XRD pattern of the samples (**e**).

The effect of heteroatom anions on the morphology and physicochemical properties of the boehmite nano-crystallites grown on the Al particles was further investigated in detail. Figure 3 shows the surface morphology and XRD patterns of the Al@AlOOH core–shell samples obtained by HTR (423 K for 5 h) in an aqueous solution (1.0 M) of NH_4_Cl, NH_4_NO_3_, and (NH_4_)_2_SO_4_, respectively. The ammonium salt solutions used for HTR of Al particles were slightly acidic at pH of 6.2–6.4. The sample prepared at the neutral pH in D.I.-water is also presented for comparison. The SEM micrographs of the Al@AlOOH resultants reveal considerably different morphological features of the boehmite nano-crystallites constructed by HTR of Al. The crystallites grown in D.I.-H_2_O display thick platelets or cuboid type 3D shapes (Figure 3a). However, the sample prepared in the presence of Cl^−^ ions exhibits extremely thin platelets (thickness = ~21 nm), constructing the Al@AlOOH core–shell with flower-like surface morphology. The average W/T aspect ratio of the crystallites was about 13.3, indicating the preferential formation of the crystallites in 2D geometry (Figure 3b). The individual γ-AlOOH platelets were developed perpendicular to the Al surface like petals constructing the flower-like overall morphologies. Similarly, the Al@AlOOH core–shell prepared in the presence of NO_3_^−^ ions also exhibited the flower-like surface morphology with thin γ-AlOOH crystallites of 2D geometry (W/T aspect ratio = 9.2). However, the Al@AlOOH core–shell developed in the presence of SO_4_^2−^ ions exhibited largely different surface morphology with a number of γ-AlOOH crystallites preferentially grown into 1D geometry. The spatial orientation of these crystallites was highly uniform, constructing an urchin-like overall morphology. The XRD results of all the Al@AlOOH samples clearly show a superposition of the characteristic diffraction patterns of Al metal and γ-AlOOH crystallites. The average crystal sizes of the resulting boehmites (samples shown in Figure 3a–d) estimated by Debye–Scherrer equation from the (020) diffraction peak was 28 nm (3a), 14 nm (3b), 15 nm (3c), and 6 nm (3d). The SEM and XRD results collectively indicate the formation of the core–shell composite architecture. These results suggest that the presence of heteroatom anions in the synthesis solution significantly affects the growth characteristics of boehmite crystallites at the Al-metal/solution interface during the HTR of Al.

### 3.3. The Effects of Solution pH on the Properties of Al@AlOOH Core–Shell Composites

**Figure 4:**
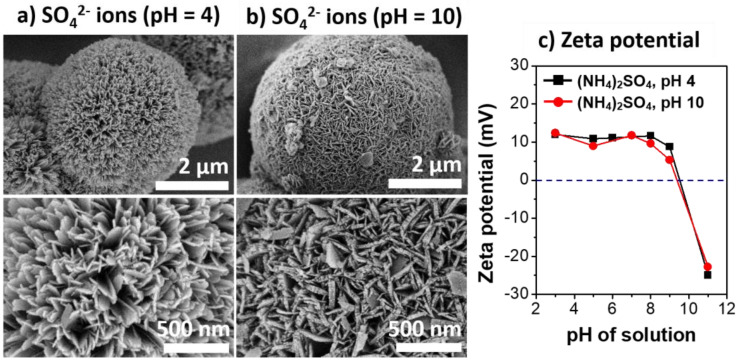
SEM micrographs of Al@AlOOH samples prepared by HTR in the aqueous solution of (NH_4_)_2_SO_4_ at pH of 4.0 (**a**) and 10.1 (**b**). The zeta potential of the Al@AlOOH measured at various solution pHs (**c**).

The effects of charge interactions of γ-AlOOH with the heteroatom anions on the growth rate and morphology of the boehmite nano-crystallites were further investigated in aqueous solutions of SO_4_^2−^ under acidic (pH = 4.0) and basic (pH = 10.1) conditions. The initial pH of aqueous (NH_4_)_2_SO_4_ solutions was adjusted by carefully adding H_2_SO_4_ or NH_4_OH, respectively. Figure 4 shows the SEM micrographs of the Al@AlOOH samples and the surface zeta-potential measured as a function of the solution pHs. The SEM micrographs reveal the large changes in the morphology of the boehmite crystallites in accordance with the change in the initial pH of the solution. In the acidic solution (pH = 4.0), the boehmite crystallites showed a preferential growth into 1D geometry. In contrast, the shape of boehmite crystallites and the surface morphology of the Al@AlOOH core–shell obtained with the basic solution (pH = 10.1) were completely different, exhibiting 2D platelets with an average W/T aspect ratio of 6.2. The surface zeta-potential of the Al@AlOOH samples indicate that the point-of-zero charge (PZC) of the surface of Al@AlOOH was reached at the solution pH of 9.5. Therefore, a positively charged γ-AlOOH shell surface can be expected in acidic solutions with pHs below 9.5. In this regime, attractive electrostatic charge interactions between γ-AlOOH surface and SO_4_^2−^ anions would occur to a significant extent. On the contrary, under basic conditions at pHs above 9.5, the charge interactions between the NH_4_^+^ cations and the negatively charged surface of γ-AlOOH can be expected. As will be discussed, these electrostatic charge interactions occur preferentially on specific crystal facets of γ-AlOOH crystallites during HTR of Al at the metal/solution interface. These interactions result in the variations in surface morphology of boehmite nano-crystallites, thereby determining the structural and physicochemical properties of the final Al@Al_2_O_3_ core–shell microstructures.

### 3.4. Physicochemical Properties of the Al@Al_2_O_3_ Core–Shell Microstructures

**Figure 5:**
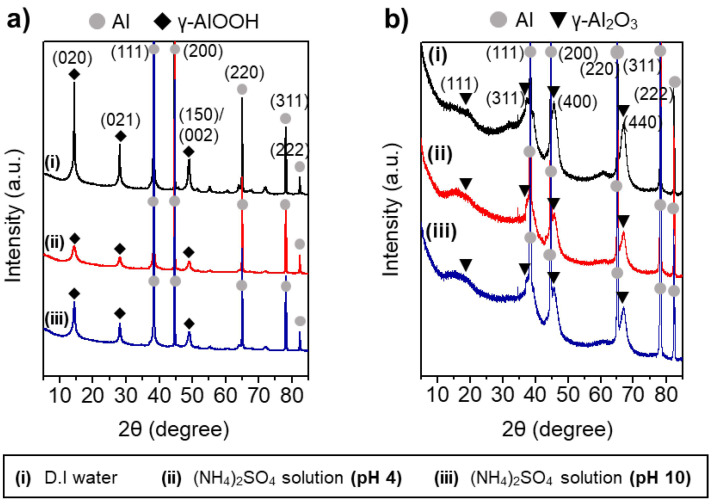
XRD patterns of the Al@AlOOH core–shell samples prepared by HTR in D.I. water and aqueous solution of (NH_4_)_2_SO_4_ at different pHs of 4.0 and 10.1 (**a**) and the Al@Al_2_O_3_ core–shell obtained by calcination of the Al@AlOOH samples at 823 K for 4 h (**b**).

**Figure 6:**
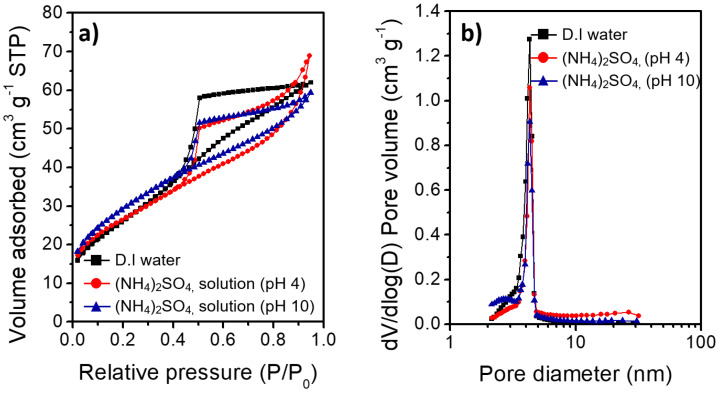
(**a**) N_2_ adsorption–desorption isotherms (**b**) Barrett–Joyner–Halenda (BJH) pore-size distribution of the Al@Al_2_O_3_ core–shell samples obtained under various synthesis conditions.

The largely different morphologies of the boehmite nano-crystallites prepared under acidic and basic conditions reflect the marked effects of the charge interactions between the heteroatom ions and γ-AlOOH surface during HTR of Al metal substrates. Figure 5 shows the XRD results of the Al@AlOOH core–shell samples and the Al@Al_2_O_3_ obtained by calcination of the Al@AlOOH core–shell in the air at 823 K for 4 h. The XRD results indicate the topotactic transformation of γ-AlOOH to porous γ-Al_2_O_3_ crystallites by the calcination treatment. The typical diffraction peaks of the γ-AlOOH disappeared after the calcination and the peaks corresponding to the γ-Al_2_O_3_ newly appeared. The peaks at 19.4, 37.6, 45.7, and 66.6° could be assigned to the diffraction on (111), (311), (400), and (440) crystal planes of γ-Al_2_O_3_ (ICSD #291495). The overall surface and core–shell morphologies of the samples were retained after calcination. The N_2_ adsorption–desorption isotherm and BJH pore size distribution of the Al@Al_2_O_3_ core–shell samples prepared in D.I.-H_2_O and the presence of SO_4_^2−^ ions at the solution pH of 4.0 and 10.1 are shown in Figure 6. The BET surface area, average pore size, and total pore volume of the samples determined from the N_2_ adsorption–desorption isotherms are summarized in Table 2. All the Al@Al_2_O_3_ core–shell samples displayed the type IV isotherm which is the characteristics of mesoporous materials. Sharp H3 type hysteresis could be seen in the P/Po range of 0.4–0.95, demonstrating the presence of parallel slit-shaped pores due to the development of the crystalline γ-Al_2_O_3_ shell on the Al@Al_2_O_3_ core–shell structure. The presence of a sharp BJH pore-size distribution peak centered at ~4 nm confirmed the uniform size of pores of the γ-Al_2_O_3_ nano-crystallites attributable to their high crystalline degree. The high BET surface area of the Al@Al_2_O_3_ samples (95–106 m^2^ g^−1^) suggests significant weight contents of the γ-Al_2_O_3_ shell in the Al@Al_2_O_3_ core–shell. The net weight amount of γ-Al_2_O_3_ in the Al@Al_2_O_3_ core–shell was estimated by considering the typical specific surface area of crystalline γ-Al_2_O_3_ (150 m^2^ g^−1^) and the density of Al metal; the value was 66 wt.%. The thickness of the Al_2_O_3_ shell estimated for an Al@Al_2_O_3_ core–shell of the diameter of 20 μm was approximately 400 nm, which was in good agreement with the thickness of the shell observed by SEM. These results collectively suggest that the unique core–shell micros-architecture consisting of an Al metal core and highly porous γ-Al_2_O_3_ shell can be constructed effectively via an interfacial HTR of Al metal substrates. The surface morphology, size, and spatial orientation of the γ-Al_2_O_3_ nano-crystallites can be modulated directly in the HTR step by introducing heteroatom ions to induce specific charge interactions between the ions and the crystal facets of boehmite nano-crystallites.

## 4. Discussion

The results obtained in this work demonstrate that unique Al@Al_2_O_3_ core–shell metal-ceramic composite microstructures can be constructed by a simple HTR of Al metal substrates. HTR of Al at the Al-metal/H_2_O interface leads to the dissolution of Al in the form of Al-hydroxides and their reconstruction into crystalline γ-AlOOH resulting in the Al@AlOOH core–shell architecture. This dissolution–reconstruction process is largely attributed to HTR temperature, presence of heteroatom ions, and pH of the solution. As can be seen in Figure 1 and Figure 2b, the HTR of Al metal particles in D.I.-water formed the core–shell composite consisting of the Al metal core and the densely grown boehmite shell. Post-calcination leads to the topotactic transformation of γ-AlOOH into γ-Al_2_O_3_ by dehydration, retaining the final core–shell architecture and surface morphologies. Therefore, the modulations in morphology, size, and structure of boehmite nano-crystallites during HTR of Al metal particles have marked impacts on the properties of the final Al@Al_2_O_3_ core–shell composites.

The phase thermodynamics of aluminum oxides predicts γ-AlOOH as the lowest energy crystal structure within the temperature and pressure conditions applied in this work [18], suggesting that the heterogeneous growth of γ-AlOOH at the Al-metal/H_2_O interface was thermodynamically driven. The crystal structure of boehmite can be described as an octahedral Al atom coordinated by four tetrahedral O atoms and two OH groups constructing an AlO_4_(OH)_2_ building unit with orthorhombic symmetry. These AlO_4_(OH)_2_ octahedrons are linked with each other by sharing O atoms along the (100) and (001) directions. The hydroxyl groups exposed to the (010) direction form interlayer hydrogen bonding constructing the typical lamellar structure of γ-AlOOH. The -OH groups can protrude from the (010) and (001) crystal planes to allow ions in the solutions to interact. First principal calculation and dynamic simulation studies predicted that the surface energy of the (010) facet of boehmite crystal is the lowest followed by the (001) and (100) facets [19,20,21]. The Gibbs–Curie–Wulff law describes that growth rates of crystal planes are proportional to the crystal surface energy; therefore, the low surface energy planes have a high tendency to be exposed in the final crystal morphology [22]. This theoretical calculation study agree with the platelet- or flake-type 2D morphologies of boehmite crystallites prepared by the conventional homogeneous synthesis approaches reported extensively in the literature. The surface morphology of the Al@AlOOH core–shell (Figure 1) prepared in this work at neutral pH (7.1) and low temperature (373 K) indicate the growth of a platelet-type 2D structure of boehmite crystallites on the Al metal surface. However, the morphology of the crystallites grown at the high temperature of 423 K resembled a cuboid-like 3D structure, that appears to reflect much-enhanced growth of the crystallites in the [010] direction at elevated temperatures.

The results shown in Figure 2 suggest that the pH of the solution significantly affected the morphology of boehmite crystallites constructed on the Al metal surface. The crystallites grown under the basic condition were not much different from those grown under a neutral condition, except that the thickness of the platelets was smaller. However, drastic changes were seen in the sample prepared in an acidic solution (pH = 3.3, HCl solution) with a number of thin nano-platelets grown on the Al surface with a W/T aspect ratio (24) much greater than those prepared under the neutral (W/T aspect ratio = 1.9) and basic conditions (W/T aspect ratio = 4.1). It is noteworthy that the net amount of boehmite crystallites formed by HTR showed no significant dependence on the solution pH, as deduced from the similar surface area and pore volume of the Al@Al_2_O_3_ samples prepared at the same temperature and time but under very different pH conditions. Intuitively, higher dissolution rates of Al atoms from the Al metal surface were expected in acidic and basic solutions considering the amphoteric nature of Al, but the similar surface area and pore volume of the samples suggested that the interfacial growth rate of boehmite crystallites was diminutively affected by solution pHs. The results suggest that the diffusion of Al species through the thickening γ-AlOOH shell layer is likely the kinetically limiting process rather than the dissolution of Al species from the Al surface for the growth of the boehmite crystallites at the interface.

The different morphologies of boehmite crystallites on Al@AlOOH core–shell samples shown in Figure 3 reveal that the presence of inorganic anions has a critical effect on the final morphology of γ-AlOOH grown on the Al surface. The zeta potential of the Al@AlOOH core–shell measured at various solution pHs (Figure 4c) showed the point of zero charge (PZC) at ~9.5, indicating that the surface of boehmite crystallites was in a positively charged state during HTR in the acidic solutions used for the samples shown in Figure 3. It can be deduced that the –OH groups protruding in the (010) and (001) directions of γ-AlOOH would be in the positively charged –OH_2_^+^ state, therefore inducing strong electrostatic charge interactions with the inorganic anions in the solutions. These charge interactions would lead to the strong adsorption of the anions (Cl^−^, NO_3_^−^, SO_4_^2−^) on the (010) and (001) facets of the γ-AlOOH, which would further lead to inhibition of crystal growth in the (010) and (001) directions. Previous theoretical calculation and experimental studies reported in the literature suggest that these inorganic ions adsorb on the (010) facet more strongly than on the (001) facet. Meanwhile, the adsorption ability of these heteroatom anions can be expected to decrease in the order of SO_4_^2−^ > Cl^−^ > NO_3_^−^, considering the charge to size ratio of the ions. It can be deduced that the thin platelet-type 2D morphology of the boehmite crystallites on the Al@AlOOH samples prepared in the aqueous solutions of HCl and HNO_3_ can be attributed to the strong adsorption of Cl^−^ and NO_3_^−^ anions on (010) facet of the boehmite crystals, resulting in its anisotropic growth preferentially towards the (100) and (001) directions. In the aqueous solution of H_2_SO_4_, it appears that SO_4_^2−^ ions strongly adsorb both on the (010) and (001) facets, leading to the preferential 1D growth of boehmite crystallites towards the (100) direction as can be seen from the urchin-like surface morphology of Al@AlOOH (Figure 3d). Adsorption of the inorganic anions on Al@AlOOH is not likely to occur in the basic solution with pH above the PZC. As can be seen clearly in the case of the Al@AlOOH samples shown in Figure 4, the 1D-type morphology of boehmite crystallites was not obtained when the solution pH was higher than the PZC, although SO_4_^2−^ anions were present in the solution. The XRD results of the Al@AlOOH and Al@Al_2_O_3_ samples (Figure 5) indicate the topotactic transformation of γ-AlOOH into porous γ-Al_2_O_3_ by calcination treatment, thereby resulting in retainment of the metal–ceramic core–shell micro-architecture. The high surface area and pore volume as well as the sharp pore size distribution of the Al@Al_2_O_3_ core–shell samples (Figure 6, Table 1) demonstrate their significant properties for applications as heterogeneous catalysts. The studies on the effects of various morphologies and exposed crystal facets of γ-Al_2_O_3_ nano-crystallites and the Al@Al_2_O_3_ core–shell microstructures on catalytic chemical reactions are currently under progress.

## 5. Conclusions

The fine control of morphology, size, and crystal structure of porous γ-Al_2_O_3_ is of significant importance in many application areas such as heterogeneous catalysts and nanomaterials. In this work, we reported a morphology and structure controlled in-situ synthesis of γ-AlOOH nano-crystallites utilizing Al metal substrates as the template and sole Al source via dissolution–reconstruction chemistry of Al at the metal/solution interface and the selective interactions of boehmite crystal facets with heteroatom anions in the solution. The Al@Al_2_O_3_ core–shell micro-architecture consisting of a Al metal core and porous γ-Al_2_O_3_ shell could be constructed by heterogeneous hydrothermal reactions of Al metal particles in aqueous solution with fine modulation of the morphologies and exposed crystal facets of γ-Al_2_O_3_ crystallites. The formation of γ-AlOOH nano-crystallites at the Al-metal/solution interface is largely affected by temperature, presence of heteroatom ions, and pH of the solution. A controlled synthesis with variation of the electrostatic charge interactions between γ-AlOOH and heteroatom anions (Cl^−^, NO_3_^−^, SO_4_^2^^−^) allows the modulation of the γ-Al_2_O_3_ crystallites morphologies and structures into 1D, 2D, and 3D nanostructures. The Al metal derived synthesis of Al@Al_2_O_3_ core–shell microarchitecture with fine modulation of Al_2_O_3_ crystallite morphologies and structure is unique and can provide significant properties and opportunities over the conventional synthesis approaches and materials. It is proposed that the unique core–shell architecture with controlled surface properties of the Al@Al_2_O_3_ metal–ceramic composites can offer significant advantageous for heterogeneous catalysts such as enhanced heat and mass transport, and high dispersion of active metal nanoparticles on the surface of Al_2_O_3_, and modulation of acid–base properties on the catalyst surface. The studies on these aspects are currently ongoing and will be reported separately.

## Figures and Tables

**Table 1:** Thermal conductivity and heat capacity of various metals and metal oxides.

Title 1	Materials	Thermal Conductivity (W m^−1^ K^−1^)	Specific Heat Capacity (J mol^−1^ K^−1^)	Reference
	Al_2_O_3_	36	78	[9]
Metal Oxides	SiO_2_	1.3	44	
	TiO_2_	8.4	57	
	Al	273	24	
Metal	Au	315	25	
	Stainless steel	13	28	[10]

**Table 2:** Surface area, average pore size, and total pore volume of Al@Al_2_O_3_ samples.

Synthesis Condition	BET Surface Area (m^2^ g^−1^)	Average Pore Size (nm)	Total Pore Volume (cm^3^ g^−1^)
D.I. Water	98	3.9	0.096
(NH_4_)_2_SO_4_ (pH = 4)	95	4.9	0.110
(NH_4_)_2_SO_4_ (pH = 10)	106	4.0	0.092

## Data Availability

The data presented in this study are available on request from corresponding author.
